# Study on Thermal Insulation Performance of Silica Aerogel Thermal Insulation Blankets

**DOI:** 10.3390/gels10110707

**Published:** 2024-10-31

**Authors:** Hao Li, Weidong Xu, Liyan Zhu, Feifei Xiao, Zhou Yu, Bentian Hao, Wei Huang, Kai Zhao

**Affiliations:** College of Field Engineering, PLA Army Engineering University, Nanjing 210007, China; lilz@aliyun.com (H.L.);

**Keywords:** aerogel, insulation, thermal conductivity, temperature

## Abstract

In this paper, the thermal insulation performance of silica aerogel was studied. Aerogel heat insulation blankets can be widely used in the military, cold storage, aerospace, automotive and other industries. The heat insulation principle of aerogel was analyzed theoretically, and the heat transfer model of aerogel was established. Experiments are designed to verify the accuracy of the model, and it is concluded that the distance between the aerogel and the target is more important for the thermal insulation effect than the thickness of the aerogel.

## 1. Introduction

With the development of infrared reconnaissance technology, traditional thermal insulation materials have been difficult to meet military needs, and thermal insulation materials can reduce the target surface temperature, so the study of new high-efficiency thermal insulation materials such as aerogel is a major trend [1,2,3,4,5].

Aerogel is a solid material with a nanoscale pore structure that has a porosity of up to 90% [6]. Because of this special void structure, it results in a thermal conductivity that can even be smaller than that of air, and the current industrialized aerogel has a thermal conductivity as low as 0.012 W/mK [7]. Aerogel can effectively isolate the surface temperature of the heat source due to its good thermal insulation effect, so as to reduce its infrared exposure symptoms and reduce the probability of being found by the infrared detector. As early as 1931, Kistler synthesized aerogel for the first time using an acidic solution of sodium silicate [8]. In 2001, Aspens Aerogel was officially established, which developed aerogel blankets, etc., and was widely used in aerospace and construction [9]. In 2015, He Yaling et al. conducted a study on the thermal conductivity model of aerogel, and pointed out that the thermal conductivity coefficient of aerogel can be fitted to synthesize the thermal conductivity coefficient of a function of material density [10], and in 2023, Xiao Junying et al. synthesized rutile titanium-nickel-yellow/silica aerogel with high NIR reflectance through the solid-phase method, which has good color rendering, low transmittance and high NIR reflectance (96.6%) [11]. Beatriz Merlilas et al. characterized the textural properties, mechanical properties and thermal conductivity of silica aerogel. The thermal conductivity of the prepared silica polyurethane foam is as low as 14 mW/m·K [12]. In 2024, Akos Szabo et al. demonstrated thermal analysis of aerogel and its vacuum form, potential uses, etc. [13]. In recent years, as the preparation process of aerogel has become more and more mature, its application has become more and more extensive, and the requirements for its thermal insulation performance have also gradually improved.

Most current scholars calculate or predict the thermal conductivity of aerogel by analyzing the aerogel itself and observing its microstructure through a scanning electron microscope, Hall measurement system, etc. [14,15,16,17]. Numerical analysis by Matteo Sambucci et al. shows that FRAB Cryogel^®^ Z system is a potential candidate to replace traditional vacuum insulators with the same thickness [18]. Cryogel^®^ Z ensures that the temperature gradient within the LNG field is lower than in the ultra-vacuum configuration (from −20% at 5 mm thickness to −18% at 20 mm thickness). Akos’ Lakatos et al. experimentally pointed out the good thermal properties of aerogel samples and graphite EPS samples. The thermal conductivity of graphite EPS is the most sensitive to temperature changes (fluctuations), varying by about 19% in the temperature range of 0 °C to 40 °C. The purpose of this paper is to establish a model of the thermal insulation effect of aerogels on the material or the environment according to the application scenario of the aerogel and verify the accuracy through experiments as a way to provide help for the practical application of aerogel materials. The structure of this paper is as follows: firstly, analyze the thermal conductivity principle of aerogel and the factors affecting its thermal conductivity; then, derive the thermal insulation model through theory; and finally, experimentally measure the thermal conductivity and thermal insulation effect of aerogel to verify the accuracy of the model. The symbols and instructions involved in this manuscript are shown in Table 1.

## 2. Results and Discussion

### 2.1. Aerogel Insulation Model

From a microscopic point of view, the heat propagation of the aerogel’s equiporous insulation material consists of [19,20,21,22,23]:

- λ-value through the solid skeleton $\lambda_{c,s}$
- λ-value through the gas particles $\lambda_{c,g}$
- λ-value through heat radiation $\lambda_{r}$
- λ-value through gas convection the terms (λconv) that have high importance in fibrous insulations $\lambda_{conv}$
- λ-value through trapped air or air layer $\lambda_{pair}$
- λ-value through holes $\lambda_{hole}$

The total effective thermal conductivity of the aerogel material can be described as:

(1) $$\begin{array}{c} \lambda_{t}=\lambda_{c,s}+\lambda_{c,g}+\lambda_{r}+\lambda_{conv}+\lambda_{pair}+\lambda_{hole} \end{array}$$

For the Knudsen effect, the pore size of the porous material is close to or less than the mean free path of the gas, and the cavity limits the movement of the gas so that the gas cannot produce convection—that is, the heat conduction is reduced. Therefore, the thermal conductivity of aerogel can be simplified as:

(2) $$\begin{array}{c} \lambda_{t}=\lambda_{c,s}+\lambda_{r}+\lambda_{pair}+\lambda_{hole} \end{array}$$

Most of the use scenarios of aerogels are wrapped around or covered above the heat source to isolate heat, and the transmission of heat includes: conduction heat transfer, convection heat transfer and radiation heat transfer. According to the law of conservation of energy:

(3) $$\begin{array}{c} Q_{in}=Q_{out}=\sum Q_{i}=Q_{1}+Q_{2}+Q_{3} \end{array}$$

$Q_{in}$: Heat absorbed; $Q_{out}$: Heat dissipated; $Q_{1}$: Radiant heat transfer; $Q_{2}$: Conduction heat transfer; $Q_{3}$: Convection heat transfer.

(1) Objects above absolute zero will produce thermal radiation, it can be said that all objects are radiating heat all the time. The object that emits heat to the outside will also continuously absorb the heat emitted by other objects around at the same time. However, thermal radiation is different from heat conduction and convection heat transfer; radiation is not only the transfer of energy and is accompanied by the transformation of the energy form, heat source and aerogel to the process of radiating heat outward. Radiation heat transfer can be calculated by the following formula [24]:

(4) $$\begin{array}{c} q=\varepsilon \sigma \left(T_{1}^{4}-T_{2}^{4}\right)=\frac{Q}{S} \end{array}$$

q is the heat flow density per unit area (w/m^2^); ε is the surface emissivity of the heat source (0~1); σ is the Stefan–Boltzmann constant; $T_{1}$ is the surface temperature (K); $T_{2}$ is the ambient temperature (K); and S is the surface area (m^2^). Therefore, $Q_{1}$ can be expressed as:

(5) $$\begin{array}{c} Q_{1}=qs=\varepsilon \sigma \left(T_{1}^{4}-T_{2}^{4}\right)S\; \end{array}$$

(2) Due to the existence of temperature differences, the heat source will transfer heat to the aerogel, and this process is called heat conduction. Even if both the aerogel and the heat source are at rest, heat transfer will be driven by the temperature gradient. In the 18th century, Blake proposed the concept of specific heat capacity, which is the basic formula of heat conduction, through a large number of physical experiments:

(6) $$\begin{array}{c} Q_{2}=cm(T_{2}-T_{1}) \end{array}$$

**Figure 1:**
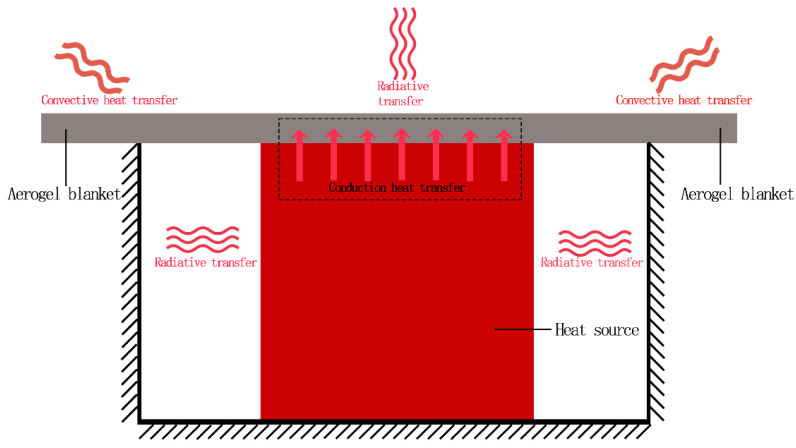
Schematic diagram of heat propagation.

In 1822, Fourier established the heat conduction equation for temperature distribution:

(7) $$\begin{array}{c} q=-k\frac{\partial u}{\partial \vec{n}},\;u_{t}=a^{2}∆u, \end{array}$$

(8) $$\begin{array}{c} a^{2}=\frac{k}{\rho c} \end{array}$$

where k, ρ, c denote the thermal conductivity, density and specific heat capacity of the material, respectively. The temperature gradient is the result of heat conduction, and solving Equation (7) requires the definition of initial conditions, boundary conditions, etc. Combined with Figure 1, the initial conditions are as follows:

(9) $$\begin{array}{c} u\left(x,\;0\right)=T\; \end{array}$$

where T is the surface temperature of the heat source.

The boundary conditions are [25]:

(10) $$\left\{\begin{array}{c} {k_{1}u_{x}|}_{x=x_{1}^{-}}={k_{2}u_{x}|}_{x=x_{1}^{+}}\\ u\left(x_{1}^{-},t\right)=u\left(x_{1}^{+},\;t\right) \end{array}\right\}$$

wherein, $k_{1},\;k_{2}$ are the thermal conductivity coefficients of the aerogel and the heat source, respectively; $x_{1}$ is the contact position of the aerogel and the heat source.

(3) Thermal convection is an important way of heat transfer, including natural convection and forced convection. The movement of the fluid between the heat source and the aerogel surface is due to the buoyancy caused by the difference in density due to the difference in temperature in the fluid, thus it is natural convection. In the absence of external disturbances such as fans, the movement of air is due to the rise of hot air and fall of cold air on the surface. Newton’s law of cooling describes this process well:

(11) $$\begin{array}{c} Q_{3}=hS(T_{2}-T_{1}) \end{array}$$

where h is the thermal convection coefficient, the value of which depends on the variables affecting convection, such as the type of motion of the fluid, the speed of motion, and so on.

### 2.2. Model Solving

**Figure 2:**
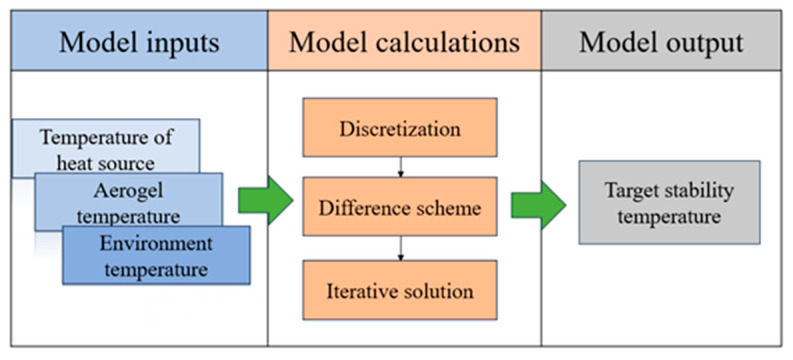
Flowchart of model solving.

In a certain scenario, given the initial temperature of the heat source, the stabilizing temperature can be found by Equations (3)–(11). However, it can be seen from the above model that it is difficult to find an analytical solution, especially since Equation (10) are functions of spacetime and can only be solved numerically. For the above model, the method of the discretization differential format iterative solution is generally adopted for calculation. The specific steps are as follows (Figure 2).

**Figure 3:**
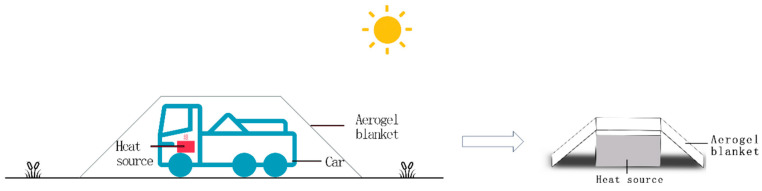
Schematic diagram of simulation solution.

To simplify the calculation, the thermodynamic analysis software can be used directly, and the results can be obtained by simulation. The numerical analysis software used in this manuscript is the thermodynamic analysis module of Solidworks software (Solidworks 2022). Assume that the background is: the field without wind conditions, just stopped driving the vehicle parked under the aerogel thermal insulation blanket. When driving or just stopping driving the vehicle, the engine is in a heating state, the temperature is up to about 100 °C, the engine is simplified as a cubic heat source and the aerogel thermal blanket is simplified as a door-type barrier, as shown in Figure 3. The meshing method we used is based on mixed curvature, with a maximum cell size of 17.8 mm and a minimum cell size of 1.9 mm.

**Figure 4:**
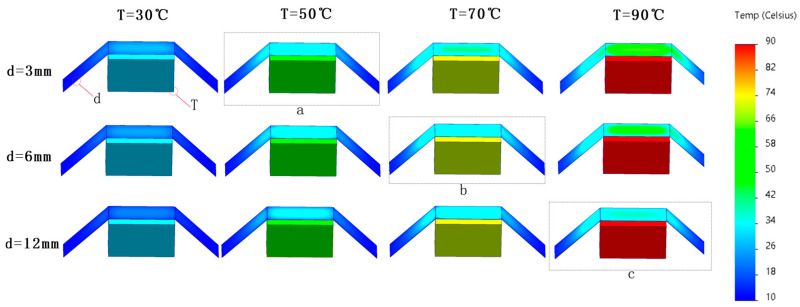
Overview of simulation calculation results.

**Figure 5:**
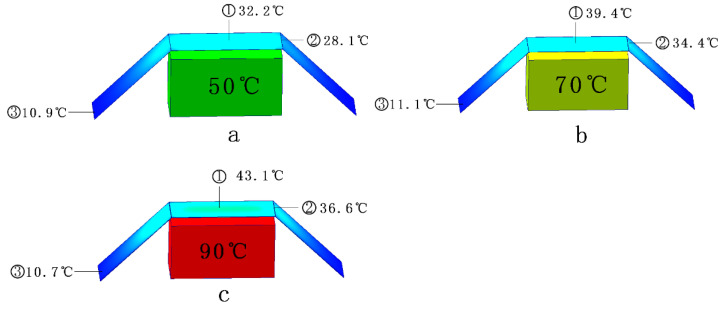
(**a**–**c**) Simulation calculation results. (**a**) Simulation results when the heat source temperature is 50 °C; (**b**) Simulation results when the heat source temperature is 70 °C; (**c**) Simulation results when the heat source temperature is 90 °C.

The setup parameters are as follows (Table 2):

The results are shown in Figure 4 and Figure 5 and Table 3:

In Figure 4, the simulated heat source is placed under the aerogel insulation blanket, and the thickness of the aerogel is d, with values of 3 mm/6 mm/12 mm, the temperature of the heat source is T, with values of 30/50/70/90 degrees Celsius, and the aerogel insulation blanket is 2 cm away from the heat source. As can be seen in Figure 5, the higher the temperature is, the darker the color of the heat source, and the aerogel is displayed, which is used to visually represent the surface temperature of the object. When the thickness of the aerogel is kept constant, with the increase in the heat source temperature, the temperature of the aerogel directly above the heat source increases, but the overall thermal insulation effect is better. When the heat source temperature reaches 90 °C, the aerogel surface temperature is only 43.1 °C (Figure 5c); when the heat source temperature is kept constant, and the thickness of the aerogel increases, the surface temperature of the aerogel decreases, which is in line with Formulas (3)–(10).

The temperatures of the heat source and the aerogel after heat exchange stabilization are given in detail in Table 3. It can be found that the thickness of the aerogel has less influence on heat exchange, and the difference between the average temperatures of 3 mm thick and 12 mm thick aerogel after stabilization is only 2.1 °C when the heat source temperature is 30 °C, and the temperature difference is only 11.7 °C even when the heat source temperature is 90 °C. Considering the application point of view, the selection of thermal insulation or anti-IR reconnaissance material is not better the thicker the aerogel thermal insulation blanket is. Combined with Figure 5, the highest temperature of the aerogel surface at the time of stabilization is at the center point directly above the heat source; when the heat source temperature is lower, the highest temperature of the aerogel is close to that of the heat source, and when the heat source temperature is higher, the difference between the highest temperature of the aerogel and the temperature of the heat source is larger, so it can be seen that the higher the temperature of the heat source is, the better the thermal insulating effect of the aerogel is.

Observing Figure 5, three results a/b/c were chosen to investigate the heat exchange law between the aerogel and the heat source. The temperature of position ➀ on the surface of the aerogel is the highest, the temperature of position ➁ is the second highest, and the temperature of position ➂ is the lowest. Regardless of the temperature of the heat source, the temperature of position ➂ stays the same as the ambient temperature, and in combination with the temperature change of position ➁ and the depth of the color, it can be speculated that there is a strong connection between the distance between the aerogel and the heat source and the stabilized temperature, and the further the distance is, the closer the stabilized temperature is to the ambient temperature.

### 2.3. Experiments on Aerogel Thermal Insulation Effect

**Figure 6:**
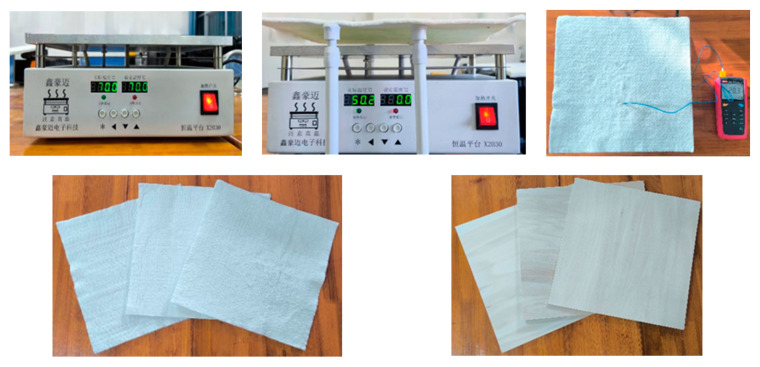
Aerogel heat insulation effect experiment. The pictures are a thermostatic table, an aerogel placed on a thermostat, a thermometer measuring the surface temperature of the aerogel, an aerogel and a whiteboard sample.

The model of the aerogel thermal insulation effect was obtained by the theoretical derivation and simulation solution in the previous section, and in order to test its accuracy, the following is verified with an experiment. Experimental design: in a closed room, using a constant temperature heating table, the aerogel insulation blanket is heated, the distance between the aerogel and the heating table is adjusted and the temperature of the aerogel is measured; the whiteboard is used for the control experiments at the same time (Figure 6). The confined space is to ensure constant temperature and pressure and isolate the environmental interference, the constant temperature heating table is used to simulate the heat source, and a conduction thermometer is used to measure the surface temperature of the aerogel and observe its real-time changes. The constant temperature heating table uses the resistance to heat and the use of a temperature control system to regulate the temperature, with an accuracy of 0.1 °C, above the thermally conductive surface for the 6061 aluminum alloy, which has a size of 30 cm × 30 cm. White boards are taken from the commonly used building materials linden boards, with moderate linden hardness and a density of 500 kg/m^3^; the de-lignification of the wood treatment improves compression, but also makes the cellulose hydroxyl groups exposed to the formation of grafting anchor points, which in turn improves its fire retardant and thermal insulation properties [26].

The room temperature of the day was 10 °C and the humidity was 50%. The experimental steps were as follows: 1. Heat the thermostatic heating table to 30 °C and place the 3 mm thick aerogel on the heating table, then record the time and temperature change after its temperature is stabilized; 2. Place the 6/12 mm thick aerogel insulation blanket and the white board as above; 3. Change the temperature of the heating table to 50/70/90 °C, place the 3/6/12 mm aerogel and the white board sequentially and record the time and temperature changes after its temperature is stabilized; 3. Record the time and temperature change; 3. Elevate the aerogel, adjust the distance between the aerogel and the heating table to 2/6/10 cm, place the 3/6/12 mm aerogel in turn and record the time and temperature changes after the temperature is stabilized.

**Figure 7:**
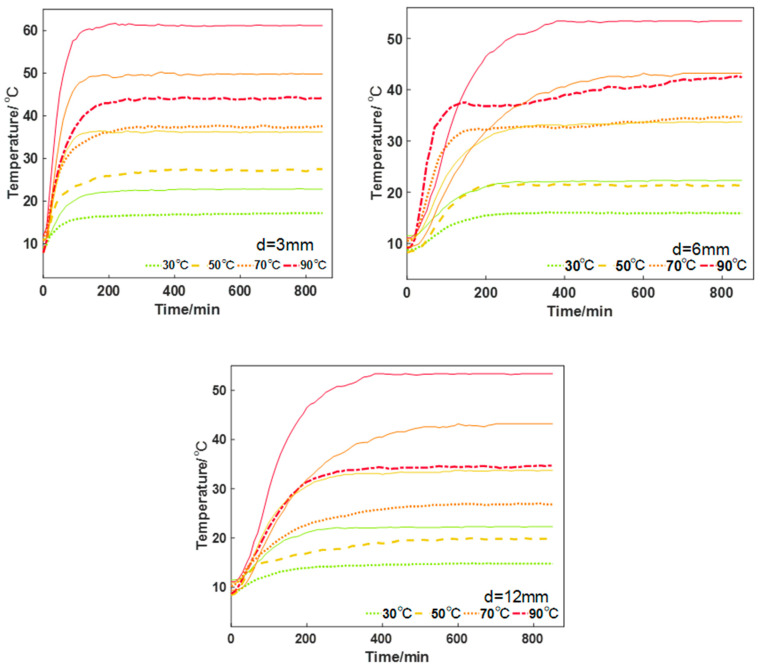
Experimental results of thermal insulation effect of 3/6/12 mm thickness aerogel and white board. The dotted line indicates the surface temperature of the aerogel, the solid line indicates the surface temperature of the whiteboard, and the lines with different colors indicate the temperature of different heat sources.

Experimental results:

Figure 7 shows the experimental results of the thermal insulation effect. In the figure, the solid line is the whiteboard temperature curve, the dotted line is the temperature curve of the aerogel and the three graphs respectively represent the 3 mm, 6 mm and 12 mm aerogel and the white board at 30/50/70/90 °C on the heating table temperature change. The overall curve is a classic “saturation curve”, with a rapid increase in temperature, and is then gradually stabilized, no longer showing a significant growth trend. Comparing the aerogel with the white board, it can be found that when the heat source is 30 °C, the stabilized temperature of the aerogel is 7 °C lower than that of the white board; when the heat source is 50 °C, the stabilized temperature of the aerogel is 10 °C lower than that of the white board; when the heat source is 70 °C, the stabilized temperature of the aerogel is 15 °C lower than that of the white board; when the heat source is 90 °C, the stabilized temperature of the aerogel is 20 °C lower than that of the white board. Therefore, it can be assumed that the aerogel has a very good heat-insulating effect, and the higher the temperature, the higher the thermal insulation effect and the better the heat insulation effect. The higher the temperature, the better the insulation effect, which is consistent with the conclusion of the previous simulation model. Regardless of whether it is the aerogel or the white board, the steepness of the curve in the first phase of the warming stage slows down as the thickness of the material increases, which indicates that a large amount of heat needs to be absorbed to realize the warming, but both materials can realize the temperature stabilization in a relatively short period of time and basically reach the thermal equilibrium in a few minutes.

**Figure 8:**
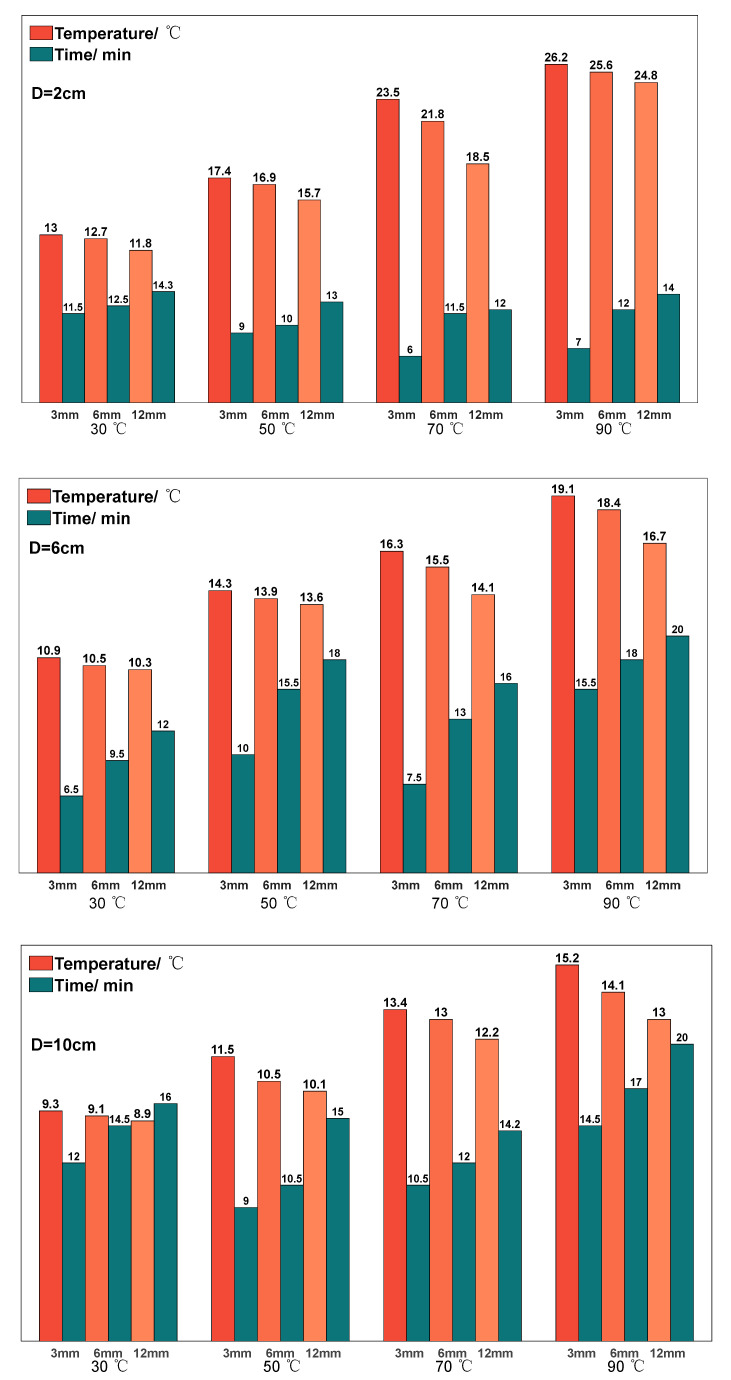
Experimental results of the thermal insulation effect obtained by adjusting the distance between the aerogel and the heat source.

The distance between the aerogel and the heat source was adjusted by raising or lowering the height of the support bar in the range of 2–10 cm, and the temperature of the aerogel after stabilization was again observed. Figure 8 shows the stabilized temperature and stabilization time of the aerogel when the temperature of the heat source is 30/50/70/90 °C and the distance is 2/6/10 cm. It can be found that the higher the temperature of the heat source, the higher the stabilized temperature and the higher the stabilized time of the aerogel. Compared to Figure 7, the stabilized temperature of the aerogel decreases dramatically in the case of having the same heat source and aerogel thickness; for example, when the temperature of the heat source is 50 °C and the thickness of the aerogel is 6 mm, the stabilized temperature of the aerogel when it is placed on the heat source directly is 21.2 °C, and the stabilized temperature of the aerogel when the aerogel is elevated by 2 cm is decreased to 16.9 °C. When the distance between the aerogel and the heat source reaches 10 cm, the stabilized temperature of the aerogel is close to the ambient temperature even if the temperature of the heat source is 90 °C, which fully demonstrates that the thermal insulation effect of the aerogel is better and the distance between the aerogel and the heat source has a greater effect on the thermal insulation effect of the aerogel. It can also be found that the thermal equilibrium time of the aerogel heat insulation blanket is more stable, and the stabilized temperature can be reached in about ten minutes, which is suitable for rapid heat insulation, cooling and other scenarios.

### 2.4. Model Testing

This paper firstly establishes the aerogel thermal insulation model and observes the aerogel thermal insulation effect through the simulation solution, and secondly through the experiment in order to check the accuracy of the model, which can be obtained by synthesizing the results of the third and fourth chapters:

(12) $$\begin{array}{c} MAE_{T_{-}mea,T_{-}sim}=3.85 \end{array}$$

**Figure 9:**
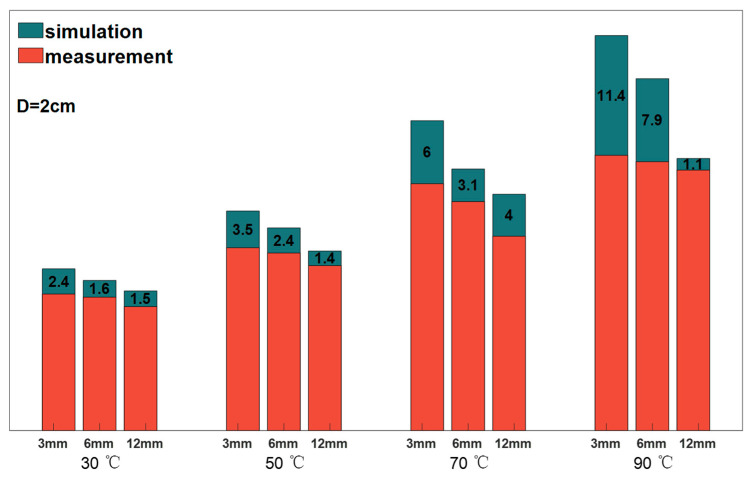
Comparison of model simulation and experimentally measured aerogel stabilization temperature.

Keeping the distance between the aerogel and the heat source at 2 cm, the stabilized temperatures of the aerogel obtained by the model simulation are shown in Table 3. The average value is taken to be compared with the experimental results, and the mean absolute error (MAE) obtained is 3.85, which is a value that indicates that the model is more accurate and can be used for the theoretical derivation related to the prediction of the temperature of the aerogel and so on. Observing Figure 9, the temperature difference between the model and the experiment is generally below 10 °C, and the larger temperature difference for the case of the heat source temperature is 90 °C with an aerogel thickness of 3 mm, and the lower the heat source temperature, the more the model agrees with the experimental results, which indicates that the model has better accuracy for the temperature of the heat source in the range of room temperature. In addition, the stabilized temperatures of the aerogels measured in the experiments were lower than those predicted by the model, which may be attributed to the following reasons: firstly, the ambient temperatures varied with the experiments, fluctuating around 10 °C; secondly, the contact thermometers used for temperature measurement had temperature loss during heat conduction [27,28,29], resulting in the measured temperatures being lower than the actual temperatures.

## 3. Conclusions

This paper first analyzes the thermal insulation principle of aerogel and derives its thermal insulation effect model, then uses simulation to solve the model, observe the thermal insulation effect of the aerogel and verify the accuracy of the model through experiments, and the following conclusions can be drawn:

1. The heating curve of aerogel is the classic “saturation curve”. Firstly, it has a rapid rising stage, then it tends to stabilize gradually and finally, it no longer shows the obvious growth trend; at room temperature, aerogel can reach the thermal equilibrium within 10 min in general.
2. The distance between the aerogel and the heat source has an important influence on the heat insulation effect. In the case of space permitting the distance between the aerogel and the heat source to increase, it is found that when this distance reaches 10 cm, even if the heat source is 90 °C, the surface temperature of the aerogel can still be close to the ambient temperature.

In summary, the excellent thermal insulation effect of an aerogel thermal insulation blanket can be widely used in petroleum, chemical, cold storage, cold chain, military, aerospace, fire, rail transportation, automotive, new energy and other industries, but there is still need to study a number of issues, such as (1) research on the cooling process of the aerogel: the aerogel may need a longer period of time to stabilize to the ambient temperature; (2) research on the stability of the aerogel, repeated heating and cooling and its thermal conductivity coefficient have a large change; (3) improving the mechanical properties of aerogel through composite technology to meet the complex scenarios in engineering applications.

## 4. Materials and Methods

**Figure 10:**
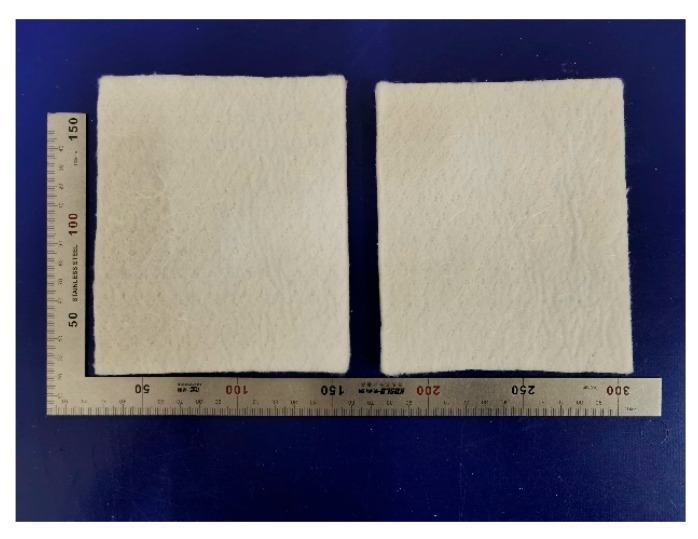
Silicon dioxide aerogel insulation blanket.

For model solving, the thermal conductivity of the aerogel needs to be determined, and we procured commercially available silica aerogel and tested its thermal conductivity using Hot Disk. The aerogel was purchased from the market in December 2023 from Namet New Materials Technology Co., LTD., Shanghai, China. Silica aerogels are poorly machinable and their practical applications are severely hampered because of their inherent brittleness and low strength, so fibers are commonly used to reinforce silica aerogels to generate composites [30]. The aerogel shown in Figure 10, a silica aerogel insulation blanket, is a combination of nano-aerogel and inorganic fibers with soft, environmentally friendly and easy-to-construct thermal insulation. For testing its thermal conductivity, the size of the sample was 12 cm × 12 cm.

**Figure 11:**
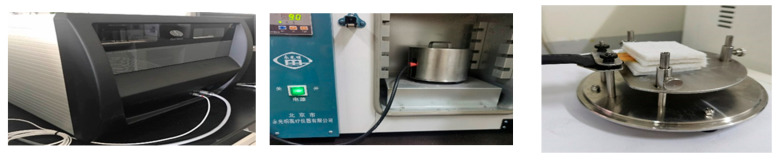
Hot Disk testing aerogel thermal conductivity.

Thermal conductivity refers to the stable heat transfer conditions: 1 m thickness of the material, 1 K difference for both sides of the surface temperature. In 1 s through the 1 square meter area of heat transfer, the Hot Disk based on the transient planar heat source method’s probe is a power output source. Also, the temperature detector, the sample and the probe are composed of sandwich mode (see Figure 11), and the probe circular coil part in the pressurized screws is directly under the two samples of the whole side of the probe clamped to ensure that there is no gap as far as possible when a stable value is reached according to the Fourier diffusion thermal conductivity equation calculations to get the thermal conductivity coefficient of the material [31].

**Figure 12:**
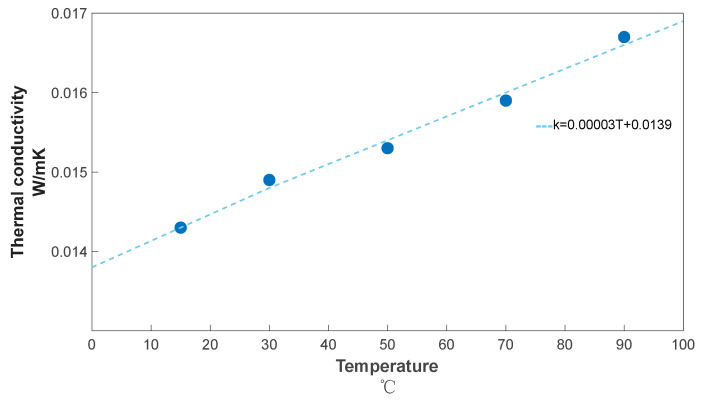
HotDisk Measurement of Thermal Conductivity of Aerogel Insulation Blanket.

The results are as follows Table 4:

The experimental results are shown in Table 4, experimentally measured thermal conductivity of the aerogel thermal insulation blanket is as low as 0.0143 W/mK (15 °C) and as high as 0.0167 W/mK (90 °C), so the thermal conductivity of the aerogel at room temperature can be considered to be around 0.015 W/mK, and the relationship between the fitted thermal conductivity and temperature is (Figure 12):

(13) $$\begin{array}{c} k=0.00003T+0.0139\; \end{array}$$

It can be seen that the temperature coefficient is of the order of 10^−5^, which indicates that the effect of the temperature on the thermal conductivity at room temperature is negligible, and the thermal conductivity can be regarded as a constant. The thermal conductivity of aerogels at room temperature is less than the air thermal conductivity of 0.02 W/mK, which is the reason why the aerogel thermal blanket has a good thermal insulation effect. Due to the Knudsen effect, the open void structure and irregular void network of the aerogel in the fabric structure greatly reduce its thermal conductivity [32].

## Figures and Tables

**Table 1:** Symbol specification table.

Symbol	Specification
$\lambda_{c,s}$	λ-value through the solid skeleton
$\lambda_{c,g}$	λ-value through the gas particles
$\lambda_{r}$	λ-value through heat radiation
$\lambda_{conv}$	λ-value through gas convection the terms
$\lambda_{pair}$	λ-value through trapped air or air layer
$\lambda_{hole}$	λ-value through holes
$Q_{in}$	Heat absorbed
$Q_{out}$	Heat dissipated
$Q_{1}$	Radiant heat transfer
$Q_{2}$	Conduction heat transfer
$Q_{3}$	Convection heat transfer
q	The heat flow density per unit area
ε	The surface emissivity of the heat source
σ	Stefan–Boltzmann constant
ρ	Material density
c	Material-specific heat capacity
T	Surface temperature of the heat source

**Table 2:** Parameter list for simulation calculation.

Properties	Values
Temperature of the heat source (T)	30/50/70/90 °C
Aerogel thickness (d)	3/6/9 mm
Environmental temperature	10 °C
Convection coefficient	0 W/m^2^·K
Heat source material	Alloy steel
Heat source emissivity	0.9
Thermal conductivity of aerogel *	0.015 W/m·K
Aerogel density	250 Kg/m^3^

(* The value of the thermal conductivity of the aerogel depends on the experimental results, as detailed in Section 4).

**Table 3:** Simulation calculation of the surface temperature of aerogel insulation blanket (°C).

	T	30 °C	50 °C	70 °C	90 °C
d					
3 mm	max	20.6	32.3	45	58.5
	min	10.4	10.9	11.5	12.3
	ave	15.4	20.9	29.5	37.6
6 mm	max	19.2	29.4	40.4	52.1
	min	10.2	10.6	11.1	11.6
	ave	14.3	19.3	24.9	33.5
12 mm	max	17.3	25.4	34.1	43.2
	min	10.0	10.2	10.4	10.7
	ave	13.3	17.1	22.5	25.9

**Table 4:** Thermal conductivity of aerogels.

Test Temperature	Mean Thermal Conductivity (W/m·K)	Thermal Conductivity-1 (W/m·K)	Thermal Conductivity-2 (W/m·K)	Thermal Conductivity-3 (W/m·K)
15 °C	0.0143	0.0143	0.0143	0.01423
30 °C	0.0149	0.0148	0.0149	0.0149
150 °C	0.0153	0.0152	0.0153	0.0154
70 °C	0.0159	0.0159	0.0159	0.0158
90 °C	0.0167	0.0165	0.0168	0.0168

## Data Availability

The original contributions presented in the study are included in the article, further inquiries can be directed to the corresponding author.
